# Evaluation of a Pilot Project to Introduce Simulation-Based Team Training to Pediatric Surgery Trauma Room Care

**DOI:** 10.1155/2017/9732316

**Published:** 2017-02-14

**Authors:** Markus Lehner, Ellen Heimberg, Florian Hoffmann, Oliver Heinzel, Hans-Joachim Kirschner, Martina Heinrich

**Affiliations:** ^1^Department of Pediatric Surgery, Dr. von Hauner Children's Hospital, Ludwig-Maximilians-University, Munich, Germany; ^2^Department of Pediatrics, University Hospital, Tuebingen, Germany; ^3^Working Group PEADSIM e.V., Department of Pediatrics, University Hospital, Tuebingen, Germany; ^4^Department of Pediatrics, Dr. von Hauner Children's Hospital, Ludwig-Maximilians-University, Munich, Germany; ^5^Department of Pediatric Surgery, University of Tuebingen, Tuebingen, Germany

## Abstract

*Introduction*. Several studies in pediatric trauma care have demonstrated substantial deficits in both prehospital and emergency department management.* Methods*. In February 2015 the PAEDSIM collaborative conducted a one and a half day interdisciplinary, simulation based team-training course in a simulated pediatric emergency department. 14 physicians from the medical fields of pediatric surgery, pediatric intensive care and emergency medicine, and anesthesia participated, as well as four pediatric nurses. After a theoretical introduction and familiarization with the simulator, course attendees alternately participated in six simulation scenarios and debriefings. Each scenario incorporated elements of pediatric trauma management as well as Crew Resource Management (CRM) educational objectives. Participants completed anonymous pre- and postcourse questionnaires and rated the course itself as well as their own medical qualification and knowledge of CRM.* Results*. Participants found the course very realistic and selected scenarios highly relevant to their daily work. They reported a feeling of improved medical and nontechnical skills as well as no uncomfortable feeling during scenarios or debriefings.* Conclusion*. To our knowledge this pilot-project represents the first successful implementation of a simulation-based team-training course focused on pediatric trauma care in German-speaking countries with good acceptance.

## 1. Introduction

Trauma is the most common cause of death in children aged one year and older and the main cause of permanent disabilities [1]. The treatment of injuries and life-threatening emergencies in children is a major cognitive challenge and an emotional burden for the treatment teams providing care in the trauma room, even in a Level 1 trauma center for children. Various studies have been able to identify massive deficiencies preclinically as well as in the pediatric surgery trauma room and in pediatric trauma care [2]. The deficits described in emergency care are found not only in the medical-specialty area, but also in the area of the so-called nontechnical skills. These nonmedical errors account for approximately 70 percent of errors according to a report published in 2000 titled “To Err is Human” [3].

Therefore, team-training concepts are increasingly being implemented in many high-risk medical fields as a tool to ensure that interdisciplinary medical care teams are best prepared for emergency situations. These courses allow medical professionals to receive training and some medical-specialty skills and learn team-oriented and behavior-oriented techniques [4].

The European division of the WHO showed that high-quality trauma care can reduce the mortality rate following trauma by up to 30 percent [1]. Numerous studies have been able to demonstrate that time delays in trauma care occur when activities are not carried out in coordinated succession [2, 5, 6].

Until now, team trainings have not been available focused on pediatric trauma room care for children with major trauma in German speaking countries.

The objective of the pilot project described in this study was, therefore, to establish interdisciplinary simulation-based team training in Germany as a tool to improve the care of trauma patients in the pediatric surgery trauma room.

## 2. Methods

The first interdisciplinary, simulation-based team training in the pediatric surgery trauma room was held in Tuebingen. The training included 14 medical doctors and 4 nurses from the medical fields of pediatric surgery, pediatric intensive care and emergency medicine, and anesthesia. All of the medical doctors were attending physicians of pediatric surgery, pediatrics, and anesthesia having more than 10 years of practice each. Two weeks before the course started, for theoretical preparation the participants received the guidelines for emergency pediatric care based on the ERC guidelines from the European Resuscitation Council 2010 (ERC). The faculty consisted of eight CRM-trained instructors from the fields of pediatric intensive care, pediatric surgery, anesthesia, and pediatric emergency medicine. Training was held in a mock trauma room in the Tuebingen Patient Safety and Simulation Center (TüPASS) of the University Hospital Tuebingen.

The course lasted 1.5 days. On the first day of the course, the participants received three hours of theoretical introduction to the topics “Trauma Room Management in Pediatric Patients” and “Errors and Patient Safety in Pediatric Emergency Care.” On the second day, participants then had a one-hour introduction to get familiarized with the simulator and the training environment. Also, skill stations focused on airway management and IO access were set up. Next, the training went through six scenarios exclusively from the field of pediatric surgery trauma room care (Table 1). The training goals were adapted to each scenario. Attention was given to ensure that all steps of the diagnostic and therapeutic algorithm of the European Pediatric Life Support (EPLS) [7] or Advanced Trauma Life Support (ATLS) courses [8] had a thematic focus in the scenarios. Here the patient was evaluated using the graduated approach following the ABCD scheme (where A = airway, B = breathing, C = circulation, and D = disability). The scenarios with each of the key medical areas and the CRM learning objectives are illustrated in Table 1. The time schedule of each training sequence was 15 minutes for the scenario with subsequently a 45-minute video-based debriefing for the participants. Therefore a higher weighting was focused on the debriefing allowing sufficient time for complete discussion of the key aspects. It was led by a two-person, interdisciplinary and multiprofessional instructor team. The ratio of medical content to CRM-related aspects was estimated to be approximately 1 : 1. As suggested by other authors, only short video sequences oriented to the learning objective were selected for the debriefing [9]. Each scenario included the active participation of 4–6 doctors and nurses as team members. The participants took on roles that corresponded to their position and their level of clinical training. The course participants who were not actively involved in the scenario observed the scenario from an adjoining room via video transmission. All participants were actively involved in at least two scenarios.

The simulation training was implemented using full-scale patient simulators: a SIMBaby (Laerdal, Stavanger, Norway) as a baby simulator and a Pediatric HAL Five Year (Gaumard, Florida, USA) as a small child simulator. The simulators were controlled from a control room outside of the simulated trauma room.

The course evaluation was conducted using anonymized pre- and postsurveys for evaluating the course and for a self-evaluation in regard to medical competency and CRM aspects. Participants were able to select from six response options on a scale between “I fully agree” = “1” and “I do not agree at all” = “6” for each item. The participants were given an anonymized code to allow for comparison between the pre- and postintervention surveys. The participants filled out the surveys and participated in the simulation training voluntarily. Statistical analysis was conducted using Microsoft Excel 2010. Significance was analyzed with Student's *t*-test; statistical significance was set at an alpha level of *p* = 0.05. All data were irreversibly made anonymous. The Ethics Committee of the Ludwig-Maximilians-University of Munich granted ethical clearance for this study, as only anonymized data were collected.

## 3. Results

A total of 18 pre- and 17 postsurveys were evaluated. The training included the participation of 14 doctors and 4 nurses from various fields. Of the doctors, 43 percent were residents. The allocation of the occupational groups was 43 percent pediatric surgery, 14 percent pediatrics, and 29 percent anesthesia. All participants treat severely injured children in their daily routine in a pediatric surgical emergency care outpatient center or an interdisciplinary emergency care outpatient center including trauma room. Of the participants, 71 percent reported having more than six years of work experience. 61 percent of the participants completed regular emergency training and 22 percent reported having already participated in simulation-based team training at least once in the past. Only 39 percent of the participants had completed an official pediatric emergency course of the established organizations of the European Resuscitation Council or the American Heart Association (ATLS [8], PALS [10], and EPLS [7]) over the course of the last two years prior to the trauma training.

Overall the individual course elements received a very positive evaluation (Table 2). The course was evaluated throughout as very realistic and relevant to the daily routine. Likewise, the detailed debriefings were evaluated as positive in the evaluation.

Individual aspects of this trauma training showed that even though this course was short, the individual participants felt there was a benefit for real care of children with critical trauma and found the feedback within the debriefings to be important and applicable to the clinical routine (Table 3). Contrary to the participants' expectations before the course, the video recordings taken during the scenarios for the debriefing were seen as slightly uncomfortable. Likewise, the participants did not feel like they were being put on display in the debriefings (Table 2).

**Figure 1:**
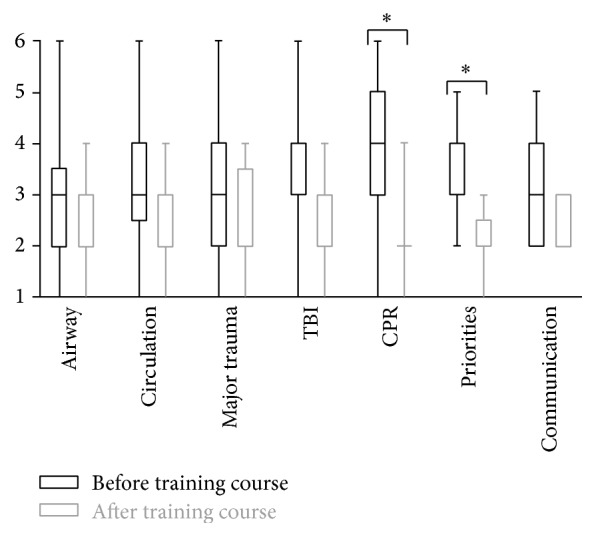
Assessment of various elements of the course pre- and posttrauma training (1 = very good, 6 = unsatisfactory, median, 25th and 75th percentiles, and span). ^*∗*^*p* < 0.001.

The participants reported of a feeling of individual improvement in almost all categories of the medical problems they worked through (Figure 1). Special medical aspects in this regard were pediatric airway management (pretrauma course: median 3, range 1–6; posttrauma course: median 2, range 1–4; not significant (ns)), circulatory problems (pretrauma course: median 3, range 1–6; posttrauma course: median 2, range 1–4; ns), polytrauma management (pretrauma course: median 3, range 1–6; posttrauma course: median 2, range 1–4; ns), management of severe head-brain trauma (pretrauma course: median 3, range 1–6; posttrauma course: median 3, range 2–4; ns), and cardiopulmonary resuscitation (pretrauma course: median 4, range 1–6; posttrauma course: median 2, range 1–4; *p* < 0.001). An improvement was likewise achieved in nontechnical skills using the example of setting priorities (pre: median 3, range 2–5; post: median 2, range 1–3; *p* < 0.001) and reliable and effective communication (pre: median 3, range 2–5; post: median 2, range 2-3; ns) (Figure 1).

## 4. Discussion

The pilot project described here was the first to introduce a simulator-based course concept focused on pediatric surgical trauma care in the German-speaking countries. For this project, the course concept of the PAEDSIM Working Group that was already tested in many trainings in pediatric emergencies involving over 1.000 participants was adapted to the special needs of a pediatric trauma emergency room [11]. In regard to the interdisciplinary character of trauma care, it seems to be necessary to integrate nonmedical aspects also, such as teamwork and communication, into established training concepts [12].

This becomes even more relevant in the time-critical emergency care of a pediatric trauma. In addition to a clear organizational structure and assignment of tasks, closed loop communication and clear team leadership are required in other course concepts [13–15]. Such was also demonstrated impressively in the scenarios practiced in this pilot project. The participants recognized for themselves the need for a clear team structure, especially in complex situations, for example, the maintenance of two patients after MVA in the trauma room (double scenario). This self-recognition is the basis for deep, experienced-based learning (deliberate practice) [16, 17]. In this respect, we believe the opportunity of having a video debriefing is an essential basis. This view coincides with the experience of other authors [18]. Participants reported that they did not feel uncomfortable with this video recording and did not feel “paraded” during scenarios. This evaluation is important to maximize the impact of training sessions and avoiding retention, in particular for simulation-based training in Germany.

In our opinion, a simulator-based course concept with a focus on CRM cannot replace the known guideline courses of medical societies (e.g., ATLS, EPLS). It is, however, a very helpful additional course focused on the important nontechnical skills during time-critical care in a large team. The “common language” of the current algorithm-oriented course formats must continue to be a basis of interdisciplinary trauma care in pediatric patients. Only those who know what is meant by the ABCDE care algorithm can function as good team members and think with foresight. To act in this way, all trainees as stakeholders in their field work as multiplicators and are responsible for the training's acceptance within their teams.

The participants' positive evaluation of the course format in regard to the relevance to daily practice and the reality of the practiced scenarios show that the instructors have selected the right scenarios. The relevance to the daily work of each participant is an important criterion of a simulation scenario developed by the PAEDSIM Working Group. According to the opinion of the authors, standard treatment of a life-threatening injury in a child is already so demanding for a multiheaded team that a conscious decision was made to exclude other devised snares, such as a power outage and other technical problems, or the presentation of rare diagnoses [12, 19].

The positive evaluation of the debriefings supports the course format with a temporary focus on the debriefing. An appropriate amount of time is needed to work through a complex incident of this type, involving the provision of care for severely injured children. Therefore, the course planners calculated 45 minutes for each debriefing. The importance of structured debriefing for the aforementioned “deep learning” of the participants is also emphasized by other authors [20, 21]. The debriefing structure proposed by Cheng et al. was effective in the course presented here as well [22]. This debriefing method, which avoids any assignment of blame and premature judgment, is a focus of the instructor training of the PAEDSIM Working Group and contributes to the good evaluation of the debriefings by the participants (Table 3).

The participants' rather average evaluation of the theoretical part calls for a revision of this section of the course. We speculate that the presentations on trauma care in pediatric patients were not adequately adjusted to the level of the participants. Due to the pilot character of the course, the majority of participants already had many years of experience in pediatric trauma care. Here a more accurate evaluation of the participants' knowledge would have been necessary at the time of course planning. Such could eventually be evaluated using an online survey distributed prior to the course. An alternative would be to reduce the amount of time for this part of the course as a way to offer more skill stations for smaller groups. Leaning on the model of the ATLS [8] courses, thematic preparation for the course would be done in private study with the help of a special manual. If participants were less experienced, an alternative would be to extend the duration of the course to two days, allowing sufficient time for interactive theoretical processing of the course content. In addition to resuscitation, pediatric trauma has been identified as an uncommon event that requires practice in managing. Furthermore to the ATLS trauma courses only a small component is devoted to pediatrics and in the EPLS courses specific performance about pediatric trauma has a low representation [23].

## 5. Summary

The objective of the present pilot project was to apply the concept of the PAEDSIM Working Group to the interdisciplinary management of pediatric trauma. We were able to show that a high-quality, simulation-based course concept can be implemented even within a narrow time frame of 1.5 days. The number of course participants is not sufficient, however, to demonstrate a subjective improvement in medical techniques and nontechnical skills based on the participants' self-evaluation. The selection of these subjective parameters as a measure of the effect of training is being viewed with increasing criticism. To evaluate the effect of simulation-based training, the personnel and organizational structure would have to remain as constant as possible and clinical quality parameters, such as the change in inner clinical care time, safety in diagnostic activities, and the quality of pediatric surgical therapy of a severely injured child, would have to be analyzed in the trauma room.

The present project is intended to serve as an impetus for further expansion of the modern and innovative educational concept of simulation-based training in pediatric surgery. This course concept is scheduled to be continued as in situ training within hospitals in the real clinical setting of interdisciplinary pediatric surgery trauma room care. This study demonstrates that simulation-based training even in pediatric trauma scenarios is feasible in an interdisciplinary setting in Germany.

## Figures and Tables

**Table 1:** The different scenarios with their respective medical and CRM priorities.

Trauma scenario	Category	Training goal	CRM goal
Hypovolemic shock in a child with blunt abdominal trauma	C	Recognition and treatment of hypovolemic shock	Reevaluation effective communication

Maintenance two patients after MVA in the trauma room (double scenario)	A B C D	Recognition and treatment of respiratory failure, rapid sequence intubation, CPR Detecting and treating a hematothorax	Team and time management prioritization Mobilization of all available resources Get help early

Tracheal tube dislocation after repositioning the patient	A B	DOPES	Avoidance of fixing errors of the tracheal tube Mobilization of all available resources

Battered child	B D	Differential diagnosis of unconsciousness CPR algorithm	Dealing with parents Double check Use of any information

Tension pneumothorax in a major injured child with thoracic contusion	A B C	Differential diagnosis of acute circulatory insufficiency Treatment of a tension pneumothorax CPR	Prioritization team leadership anticipation

Traumatic brain injury (TBI) with secondary deterioration and seizure following sledge accident	D	Treatment of TBI and seizure neuroprotection	Prioritization (diagnostic procedures versus surgical care)

A = airway, B = breathing, C = circulation, D = disability; CRM = crisis resource management; CPR: cardiopulmonary resuscitation. DOPES: D = displacement (tube), o = Obstruction (tube), P = pneumothorax, E = equipment failure, S = stomach pressure; MVA = motor vehicle accident.

**Table 2:** Evaluation of the individual course elements (1 = very good, 6 = unsatisfactory, and *n* = number of participants).

Parameter	1	2	3	4	5	6
Overall impression	14	3	—	—	—	—
Lessons (CRM + acute trauma care, emergencies)	2	2	9	4	—	—
Realism of scenarios	9	6	1	1	—	—
Relevance of the scenarios for the practice	12	3	2	—	—	—
Debriefings	11	4	—	1	—	

**Table 3:** Individual marks of the course elements (*n* = number of participants).

Parameter	I totally agree	I agree	I tend to agree	I tend to disagree	I do not agree	I do not agree at all
In this course I got benefit for my clinical practice?	13	4	—	—	—	—
The feedback from the instructors is useful for my clinical practice?	10	7	—	—	—	—
I felt uncomfortable with video recordings during the scenarios.	—	1	—	—	7	9
I feel “paraded” during scenarios.	—	—	—	2	2	14
